# Genetic variation and phylogenetic analysis of Indonesian indigenous catfish (baung fish) based on mitochondrial *12S rRNA* gene

**DOI:** 10.14202/vetworld.2021.751-757

**Published:** 2021-03-24

**Authors:** Rini Widayanti, Ken Ayik Kusumaastuti, Joana Martha Novi, Fadila Khairuna Adani, Catrine Relia Patrecia Gultom, Ayuning Devina Prastiti, Herjuno Ari Nugroho, Suhendra Pakpahan

**Affiliations:** 1 Department of Biochemistry and Molecular Biology, Faculty of Veterinary Medicine, Universitas Gadjah Mada, Yogyakarta, Indonesia; 2 Research Center for Biology, Indonesian Institute of Sciences (LIPI), Jl. Jakarta-Bogor KM 46, Cibinong, West Java, Indonesia

**Keywords:** *12S ribosomal RNA* gene, baung fish, *Hemibagrus nemurus*, Indonesian catfish, phylogenetic

## Abstract

**Background and Aim:**

Baung fish is an essential commodity in Indonesia; however, few studies have explored the genetic diversity of Indonesian catfish. Thus, this study aimed to analyze the genetic variation and phylogenetic relationships among Indonesian catfish based on the mitochondrial *12S ribosomal RNA* (*rRNA*) gene.

**Materials and Methods:**

In total, 28 catfish were collected from nine rivers in seven provinces and from the Indian Ocean. Catfish genomes were obtained from epaxial and hepaxial muscle samples. The mitochondrial *12S rRNA* gene was amplified by polymerase chain reaction using a pair of primers (Baung12SF and Baung12SR). The *12S rRNA* sequences were analyzed using MEGA X to determine genetic variation and phylogenetic relationships.

**Results:**

In total, 178 variation sites in the *12S rRNA* gene were substituted among Indonesian catfish. The genetic distance between all Indonesian catfish samples was 0.1-16.0%. The closest genetic distance was between MP and PM catfish, whereas the farthest genetic distances were between BF and EM and PD and EM. For the entire population, based on mean diversity calculations, the number of base substitutions per site was 0.08.

**Conclusion:**

Indonesian catfish were divided into four clades based on the *12S rRNA* gene. The catfish MP, KR, PM, MS, BB, and KS were grouped with *Hemibagrus nemurus*, the catfish EM was grouped with *Mystus vittatus*, the catfish BSBJ was grouped with *Pangasius pangasius*, and the catfish PD and BF were grouped with *Netuma thalassina*.

## Introduction

Indonesia is a country with enormous biodiversity (i.e., “mega biodiversity”). For example, around 16% of the world’s fish species are found in Indonesia, and 2000 of these 7000 fish species are freshwater fish. Thus, the freshwater fish population of Indonesia is second only to that of Brazil [1]. Baung fish (*Hemibagrus nemurus*) is an essential commodity in Indonesia because it is widely consumed and contains essential nutrients. For example, baung fish are a valuable source of protein, lipids (with large amounts of omega-3, omega-6, monounsaturated fatty acids, docosahexaenoic acid, and eicosapentaenoic acid), minerals, albumin, and antioxidants [2-4].

The distribution of baung fish is relatively wide in the islands of Java, Sumatra, and Kalimantan [5]. In different regions of Indonesia, this fish are known by other names such as Duri, Baon (Malay), Bawon (Betawi), Senggal or Singgah (Sunda), Tagih or Tageh (Java), and Tiken bato (Central Kalimantan). Iqbal [6] reported that 60 species of baung fish exist in Indonesia, three of which are found in the Hutan Rawa Gambut Merang Kepayang Banyuasin, South Sumatra. These types are a beringit fish (*Mystus singaringan*) two types of baung fish (*Hemibagrus hoevenii* and *Bagroides macropterus*).

The genetic markers of baung fish have been studied to identify species and preserve genetic resources; however, given the size of the aquaculture industry in Indonesia, relatively few studies have been conducted [7]. The diversity of the nucleotides of each species can be used as genetic markers, which can be used to construct phylogenetic trees and complement current molecular data that are currently lacking [8,9]. Mitochondria are membrane-bound cell organelles that generate most of the chemical energy needed to power biochemical reactions in the cell. Mitochondrial DNA contains 37 genes, all of which are essential for normal mitochondrial function. Transfer RNA (tRNA) and ribosomal RNA (rRNA) are types of RNA that help assemble amino acids into functioning proteins. The *12S* and *16S rRNA* mitochondrial genes are relatively conserved; they have evolved more slowly than the mitochondrial genome as a whole and can be used as genetic markers for the identification of species and in forensic investigations [10-12].

To date, few studies have explored the genetic diversity of Indonesian catfish. Thus, the present study aimed to characterize Indonesian catfish from different provinces (those known as baung fish by local people) and to determine the diversity among them using *12S rRNA* gene sequences and comparisons with the available GenBank sequence. Moreover, the genetic variability of the *12S rRNA* gene in catfish was measured to determine the variation and relationships among Indonesian catfish from different regions.

## Materials and Methods

### Ethical approval

This study was approved by the Animal Ethics Committee for using Animal and Scientific Procedures in Faculty of Veterinary Medicine, Universitas Gadjah Mada, Indonesia.

### Study period and location

The collection of catfish samples was carried out from 2017-2020, but for the study of Genetic variation and phylogenetic analysis of Indonesian indigenous catfish (baung fish) based on mitochondrial *12S rRNA* gene, it was conducted from January to September 2020 in the Laboratory of Biochemistry and Molecular Biology, Faculty of Veterinary Medicine, Gadjah Mada University.

### Catfish collections

Baung fish DNA was obtained from 28 samples of the epaxial and hepaxial muscles of fish from various rivers in Indonesia and from the Indian Ocean. Table-1 shows the origin, number, and code of the baung fish. All individuals were identified based on morphological characteristics and sample tissues were preserved in RNAlater buffer (Qiagen). The catfish samples in this study were considered to be unrelated genetically because they were taken individually from the rivers and ocean. Catfish were collected from the ocean to determine the relationship and genetic diversity between river catfish and sea catfish.

**Table-1:** Origin and number of Indonesian catfish.

River/Sea	Province	Number	Sample CODE
Progo river	Central Java	3	PM1, PM2, PM3
Elo river Bengawan Solo river	Central Java Central Java	2 3	EM1, EM2 BSBJ1, BSBJ2, BSBJ3
Kampar river	Riau	3	KR1, KR2, KR3
Musi river	South Sumatra	3	MP1, MP2, MP3
Mahakam river	East Kalimantan	3	MS1, MS2, MS3
Kapuas river	West Kalimantan	2	KS1, KS2
Martapura river	South Kalimantan	3	BB1, BB2, BB3
Bomberay river	West Papua	4	BF1, BF2, BF3, BF4
Indian Ocean	Yogyakarta	2	PD 1, PD2

### DNA extraction and *12S rRNA* gene amplification

The total DNA of catfish was extracted using a gSYNCTM DNA Mini Extraction Kit (Geneaid Biotech Ltd., Taiwan) following the manufacturer’s instructions and then stored at −20°C until use. The *12S rRNA* fragments of the target region were amplified by polymerase chain reaction (PCR) using a pair of primers: Baung12SF: 5′-TAA CAC TGA AGA TGT TAA GA-3′ and Baung12SR: 5′-TAG CTA AAT CAT GAT GCA AA-3′. The PCR reaction was conducted in a total volume of 50 μL, comprising 25 μL of master mix (Kapa2G ReadyMix, 1^st^ Base), 2 μL of DNA template, 1 μL (10 pmol) of each primer, and 21 μL of distilled water. Reaction cycles in an Infinigen Thermocycler comprised an initial denaturing step at 94°C for 5 min, followed by 35 cycles at 94°C for 30 s, 41°C for 45 s, and 72°C for 90 s, with a final extension at 72°C for 5 min. DNA amplifications were confirmed by 1% agarose gel electrophoresis with a 100 bp DNA ladder (Genaid) used for genotyping.

### Sequences and phylogenetic analysis

All purified PCR products were sequenced directly by 1^st^ Base Sequencing INT using forward and reverse primers. The fragments of forward and reverse *12S rRNA* gene sequences were aligned using ClustalW and edited, and then, multiple alignments were performed with data linked to *H. nemurus* and other catfish from the NCBI database. Fragments of the *12S rRNA* gene were analyzed for 956 nucleotides. Genetic distance was determined using the Kimura two-parameter method and phylogenetic relationships were assessed through the neighbor-joining (NJ) method usingMEGA X version 10.1 (https://www.megasoftware.net) [13]. The bootstrap method for genetic distance analysis included 1000 replicates. A phylogenetic tree was constructed based on *12S rRNA* sequences, and catfish sequences from other countries were used to reveal relationships and clusters among catfish. To construct the phylogenetic tree and determine relationships among catfish, the sequences of comparison species were obtained from the NCBI database: *H. nemurus* (KJ573466.1), *Mystus cavasius* (KU870465.1), *Pangasius pangasius* (KC572135.1), *Pangasianodon gigas* (AY762971.1), *Arius arius* (KX211965.1), and *Netuma thalassina* (MG587041.1).

## Results

### Genetic variation of Indonesian catfish based on the *12S rRNA* gene

The *12S rRNA* gene had a length of 959 bp and was located between the *tRNA-Phe* gene and the *tRNA-Val* gene. The amplified DNA fragments were 1309 bp in length and comprised *tRNA-Phe* (46 bp), *12S rRNA* (956 bp), *tRNA-Val* (72 bp), and *16S rRNA* (235 bp). The average respective percentage of nucleotide T, C, A, and G from each group sample was as follows: PM (22.8%, 24.9%, 31.6%, and 20.7%); EM (22.9%, 23.6%, 32.9%, and 20.6%); BSBJ (21.9%, 25.6%, 31.7%, and 20.9%); BF (20.2%, 27.8%, 30.6%, and 21.4%); KR (22.9%, 24.8%, 31.6%, and 20.7%); MP (22.8%, 24.9%, 31.7%, and 20.6%); MS (23.1%, 24.6%, 31.8%, and 20.6%); KS (22.9%, 24.8%, 31.6%, and 20.7%); BB (23.0%, 24.7%, 31.4%, and 20.9%); and PD (20.0%, 27.9%, 31.2%, and 20.9%).

**Figure-1:**
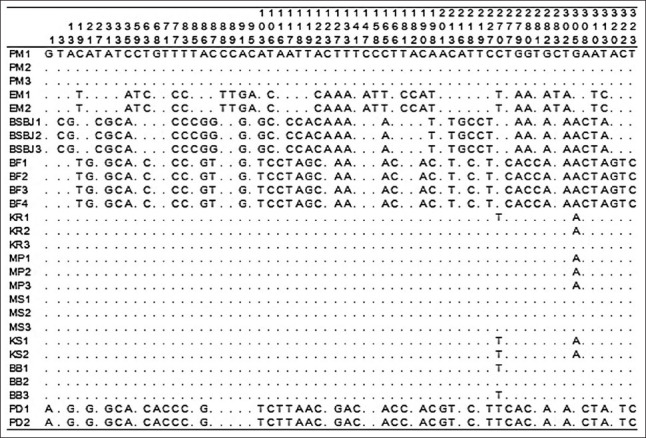
Polymorphic sites in *12S rRNA* gene of Indonesian catfish from site 1 to 323. Identity with the first sequences is denoted by a dot.

**Figure-2:**
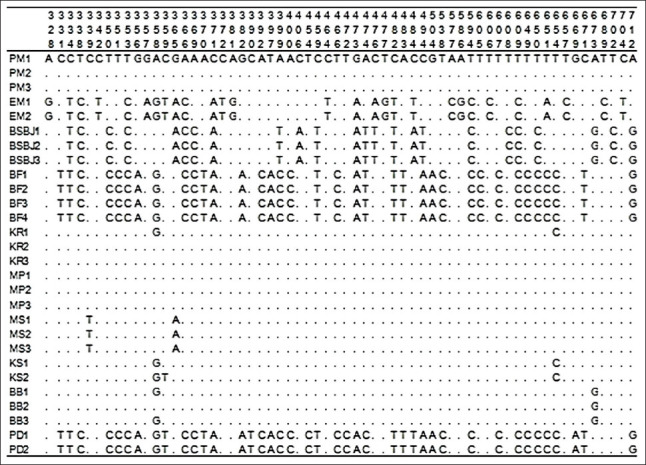
Polymorphic sites in *12S rRNA* gene of Indonesian catfish from site 328 to 712. Identity with the first sequences is denoted by a dot.

**Figure-3:**
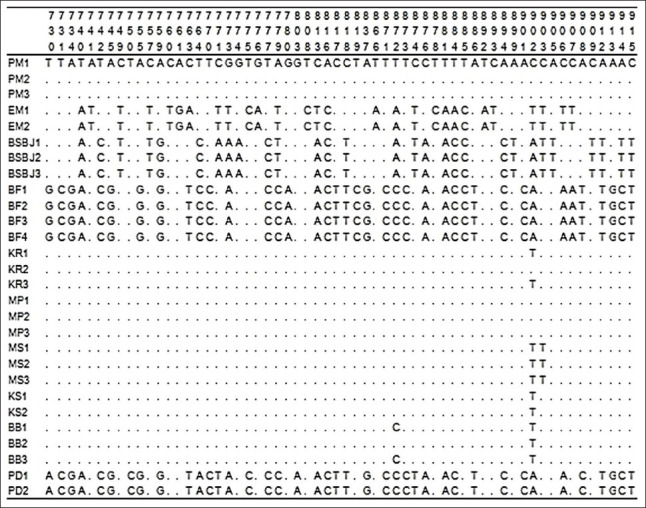
Polymorphic sites in *12S rRNA* gene of Indonesian catfish from site 730 to 915. Identity with the first sequences is denoted by a dot.

Among all samples, 178 variation sites existed in the *12S rRNA* gene, which were nucleotides substituted among the Indonesian catfish, but deletions and insertions were not found. The alignment was conducted with ClustalW based on comparisons with the PM1 sample sequence. The homology of each sample against the PM1 sample is indicated by a dot in Figures-1-3. Alignment of all sequences indicated that genetic variation existed among Indonesian catfish samples. The catfish EM, BSBJ, BF, and PD had high variation relative to the catfish PM. Several unique variation sites were identified that could serve as genetic markers for populations: EM sites 59, 372, 464, 477, 577, 586, 606, and 651; BSBJ sites 27, 118, 119, 216, 219, 406, 476, 484, 494, and 608; BF sites 116 119, 320, 730, 780, and 907; MS site 339; and MP sites 1, 68, 71, 116, 127, 141, 201, 390, 454, 472, 474, 730, 750, and 764 (Figures-1-3).

### Genetic distance analysis based on *12S rRNA* gene sequences

Baung fish from Kalimantan (MS, KS, and BB), Sumatra (MP and KR), and Java (PM) were identified as *Hemibagrus* spp. with a genetic distance of 0.1-6.0% (Table-2). Catfish EM and BSBJ were identified as *Mystus* spp. and *Pangasius* spp., respectively. Fifteen sites were found in the nucleotide variation of the *12S rRNA* gene that could be used as genetic markers among Indonesian catfish. Genetic markers distinguished baung fish from the Bomberay River, West Papua (BF1, BF2, BF3, and BF4), which were identified as *Netuma* spp. with a genetic distance of 5.2%. Fifty sites were found in the nucleotide variation that could be used as genetic markers for fish samples from the Bomberay River. The genetic distance between all samples of Indonesian catfish was 0.1-16.0%.

**Table-2:** Estimates of evolutionary divergence over sequence pairs between groups.

	PM	EM	BSBJ	BF	KR	MP	MS	KS	BB	PD
PM										
EM	0102									
BSBJ	0106	0116								
BF	0141	0161	0103							
KR	0002	0102	0106	0139						
MP	0001	0103	0105	0139	0002					
MS	0004	0100	0104	0146	0005	0005				
KS	0006	0100	0109	0139	0003	0005	0008			
BB	0005	0101	0107	0141	0005	0006	0007	0006		
PD	0151	0161	0116	0047	0151	0153	0157	0148	0150	

After averaging across all sequence pairs between groups, the number of base substitutions per site was calculated for the entire population as 0.08 (Table-2). Genetic distances were obtained by bootstrapping (1000 replicates) and analyses were conducted using the Kimura two-parameter model [14]. All ambiguous positions were removed for each sequence pair (i.e., the pairwise deletion option). Although the genetic distances between the Indonesian catfish samples in this study were from 0.001 to 0.161, the closest genetic distance was between catfish MP to PM, whereas the farthest genetic distances were between catfish BF and EM and PD to EM.

### Phylogenetic relationships of Indonesian catfish

**Figure-4:**
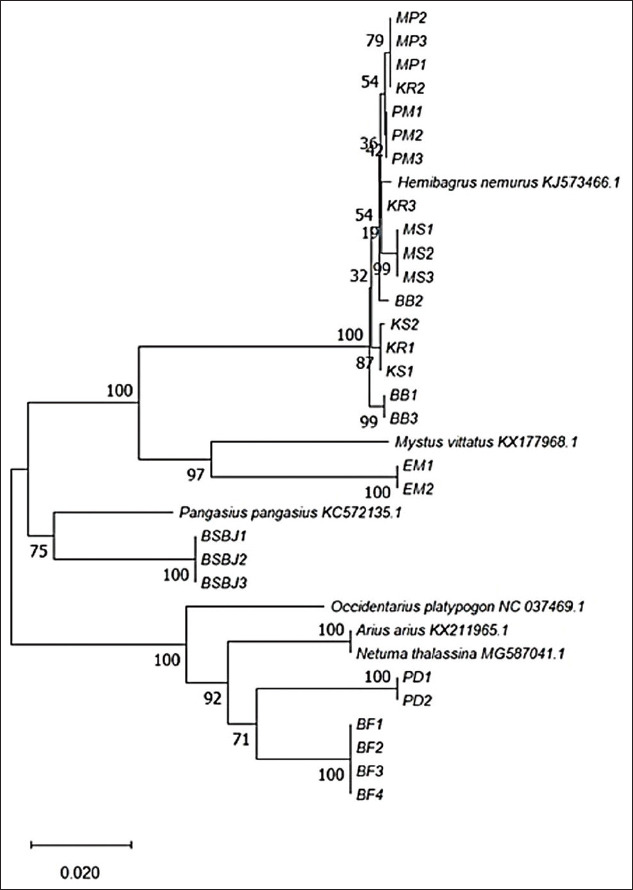
Phylogenetic relationship of Indonesian catfish based on *12S rRNA* gene sequences.

A phylogenetic tree of Indonesian catfish and other catfish from the NCBI database was constructed using the NJ method [15]. The optimal phylogenetic tree (sum of branch lengths=0.38391001) is shown in Figure-4. The percentage of replicate trees in which the associated taxa clustered together in the bootstrap test is shown next to the branches [16]. The tree is drawn to scale, with its branch lengths in the same units as those of the evolutionary distances used to infer it. The evolutionary distances are shown as the number of base substitutions per site. In total, 34 nucleotide sequences were analyzed. After all ambiguous positions were removed for each sequence pair, 970 positions existed in the final dataset. Evolutionary analyses were conducted in MEGA X [13].

The *12S rRNA* nucleotide sequence was used to examine the phylogenetic relationship among all Indonesian catfish samples and some catfish from other countries (Figure-4). First, alignment was performed in ClustalW, and then forward and reverse sequences were edited in MEGA X. A phylogenetic tree containing 28 Indonesian catfish was constructed. In this tree, the Indonesian catfish were divided into four clades with the catfish from other countries. The catfish MP, KR, PM, MS, BB, and KS were grouped with *H. nemurus*, the catfish EM was grouped with *Mystus vittatus*, the catfish BSBJ was grouped with *P. pangasius*, and the catfish PD and BF were grouped with *N. thalassina*. These four groups were supported by bootstrapping at approximately 75-100% NJ.

## Discussion

### Determining the species of Indonesian catfish based on the *12S rRNA* gene

Throughout their distribution area in Indonesia, *Hemibagrus* spp. are the fish most widely consumed as food. Thus, species identification is important for the sustainable use of this species complex [17]. Much of the biodiversity in the Indonesian archipelago has yet to be identified and/or characterized including catfish. Indeed, local Indonesian people typically use the same name for all types of catfish. Morphologically determination of species of catfish is difficult because they are highly similar in this respect. Thus, the genetic analysis provides more accurate information regarding the diversification and evolutionary relationships among species [18,19]. Such analysis is vital as catfish are found throughout the fresh and brackish waters of Asia and Africa, with more than 200 species known to exist in 17 genera, making catfish one of the largest fish families [20].

Mitochondrial DNA is popular as a target for species identification and the study of genetic diversity because it includes more mitochondrial DNA than nuclear DNA, has high variation, and lacks recombination [21]. Based on previous research by Megarani *et al*. [22], Indonesian catfish can be divided into five clades based on the *Cyt B* gene: The *H. nemurus* and *Hemibagrus wyckioides* (family Bagridae) group; the *Sperata seenghala* and *Hemibagrus spilopterus* (family Bagridae) group; the *Pseudolais pleurotaenia* (family Pangasiidae) group; the *M. cavasius* (family Bagridae) group; and the *Potamosilurus latirostris* (family Ariidae). Syaifudin *et al*. [23] identified freshwater fish in South Sumatra, such as baung (*H. nemurus*), beringit (*M. singaringan*), gabus (*Channa striata*), serandang (*Channa pleurophthalma*), and sepat (*Trichogaster* spp.), using the *Cytochrome C oxidase subunit I* (*COI*) *mtDNA* sequence; the *COI* *mtDNA* gene can be used to differentiate fish at the species level and shows effective and accurate species relatedness. Thus, both the *12S rRNA* and *COI mtDNA* genes are recommended for the identification and analysis of genetic diversity between species [11,24].

The present study used a similar sample to that researched by Megarani *et al*. [22], but one group differed, namely, the PD sample. The type of mitochondrial gene studied also differed between the two studies. Here, all sequences of the *12S rRNA* gene were blasted in the NCBI database; the results indicated that Indonesian catfish comprise four groups: *H. nemurus*, *M. vittatus*, *P. pangasius*, and *N. thalassina/A. arius*.

### Phylogenetics and phylogeographics of Indonesian catfish

Based on research by Dodson *et al*. [25], the biogeographical history of Southeast Asia contributed to extensive admixture during the Pleistocene low sea-levels of genetic groups of an obligate the river catfish that isolated during periods of high sea levels. In this study, one type of catfish was taken directly from the Indian Ocean, that is, catfish PD. The *12S rRNA* sequence analysis showed that this catfish had a close genetic relationship with catfish BF that originated from Papua Island, and these two catfish had a close genetic relationship with *N. thalassina*.

*H. nemurus*, allegedly from Southeast Asia, has previously been reported to have broad genetic subdivisions based on molecular phylogenetic analysis and phylogeography [17,23,25]. The results of the present study support those of previous studies, that is, that many species of Bagridae exist in Indonesia (about 60 species). Four species were identified here from various Indonesian islands, namely, the species *H. nemurus*, *M. vittatus*, *P. pangasius*, and *N. thalassina* (or *A. arius*). Most samples were of *H. nemurus*, which originated from the islands of Sumatra, Java, and Kalimantan (namely, catfish MP, KR, PM, MS, KS, and BB). By contrast, catfish samples from Papua and the Indian Ocean belonged to *N. thalassina* or *A. arius*. The results of this grouping were supported by high bootstrap values of 75-100% NJ. Therefore, Indonesian catfish species and even subspecies can be identified and characterized based on phylogenetic analysis, which could help to successfully conserve species.

## Conclusion

Indonesian catfish were divided into four clades based on analysis of the *12S rRNA* gene. The catfish MP, KR, PM, MS, BB, and KS were grouped with *H. nemurus*, the catfish EM was grouped *M. vittatus*, the catfish BSBJ was grouped with *P. pangasius*, and the catfish PD and BF were grouped with *N. thalassina* and *A. arius*.

## Authors’ Contributions

RW and SP designed the research and collected Indonesian catfish samples for this study. SP, KAK, JMN, FKA, CRPG, ADP, and HAN conducted research in the laboratory. RW and SP analyzed the data and wrote the manuscript. All authors have read and approved the final manuscript.
